# Posttraumatic Stress Disorder Symptoms and Cardiovascular and Brain Health in Women

**DOI:** 10.1001/jamanetworkopen.2023.41388

**Published:** 2023-11-02

**Authors:** Rebecca C. Thurston, Karen Jakubowski, Yuefang Chang, Minjie Wu, Emma Barinas Mitchell, Howard Aizenstein, Karestan C. Koenen, Pauline M. Maki

**Affiliations:** 1 Department of Psychiatry, University of Pittsburgh, Pittsburgh, Pennsylvania; 2 Department of Epidemiology, University of Pittsburgh, Pittsburgh, Pennsylvania; 3 Department of Psychology, University of Pittsburgh, Pittsburgh, Pennsylvania; 4 Department of Neurosurgery, University of Pittsburgh, Pittsburgh, Pennsylvania; 5 Department of Epidemiology, Harvard T.H. Chan School of Public Health, Boston, Massachusetts; 6 Department of Psychiatry, University of Illinois at Chicago, Chicago; 7 Department of Obstetrics and Gynecology, University of Illinois at Chicago, Chicago

## Abstract

**Importance:**

Posttraumatic stress disorder (PTSD), cardiovascular disease (CVD), and Alzheimer disease are major public health issues, particularly for women. The implications of PTSD for cardiovascular and brain health for women is poorly understood.

**Objective:**

To assess whether PTSD symptoms among midlife women are associated with carotid intima media thickness (IMT), an indicator of carotid atherosclerosis; brain white matter hyperintensity volume (WMHV), an indicator of brain small vessel disease; and cognitive performance and to test a modifying role of the *APOE*ε4 genotype.

**Design, Setting, and Participants:**

In this cross-sectional study, participants were enrolled between 2016 to 2021 and completed questionnaires (PTSD Checklist–Civilian Version), physical measures, phlebotomy, neuropsychological testing, a carotid ultrasonographic examination, and 3-Tesla brain magnetic resonance imaging. Participants included community-based women ages 45 to 67 years without a history of CVD, stroke, or dementia. Data were analyzed from July 2022 to September 2023.

**Exposures:**

PTSD symptoms.

**Main Outcomes and Measures:**

Outcomes of interest were associations of PTSD symptoms with carotid IMT, brain WMHV, and cognition, assessed in linear regression models. Interactions by *APOE*ε4 were tested. Covariates included age, race and ethnicity, education, and CVD risk factors.

**Results:**

Among 274 participants (mean [SD] age, 59.03 [4.34] years; 6 Asian participants [2.2%]; 48 Black participants [17.5%]; 215 White participants [78.5%]; 5 multiracial participants [1.8%]), 64 participants (24.71%) were *APOE*ε4 genotype carriers. Higher PTSD symptoms were associated with greater carotid IMT (multivariable β = 0.07 [95% CI, 0.01 to 0.13]; *P* = .03). Associations of PTSD symptoms with neurocognitive outcomes significantly varied by *APOE*ε4 status. Among women with *APOE*ε4, PTSD symptoms were associated with greater whole-brain WMHV (β = 0.96 [95% CI, 0.30 to 1.63]; *P* = .009), periventricular WMHV (β = 0.90 [95% CI, 0.24 to 1.56]; *P* = .02), deep WMHV (β = 1.21 [95% CI, 0.23 to 2.20]; *P* = .01), and frontal WMHV (β = 1.25 [95% CI, 0.05 to 2.45]; *P* = .04), as well as with poorer cognition, specifically attention and working memory (β = −3.37 [95% CI, −6.12 to −0.62]; *P* = .02), semantic fluency (β = −6.01 [95% CI, −10.70 to −1.31]; *P* = .01), perceptual speed (β = −12.73 [95% CI, −20.71 to −4.75]; *P* = .002), and processing speed (β = −11.05 [95% CI, −17.80 to −4.30]; *P* = .002) in multivariable models.

**Conclusions and Relevance:**

In this cross-sectional study of midlife women, greater PTSD symptoms were associated with higher carotid atherosclerosis and, among women who were *APOE*ε4 carriers, greater brain small vessel disease and poorer cognitive performance. These findings point to the adverse implications of PTSD symptoms for cardiovascular and neurocognitive health among women in midlife, particularly for women who are *APOE*ε4 carriers.

## Key Points

**Question:**

Are posttraumatic stress disorder (PTSD) symptoms associated with poorer cardiovascular and neurocognitive health among midlife women, and do these associations vary by *APOE*ε4 status?

**Findings:**

In this cross-sectional study of 274 midlife women, women with higher PTSD symptoms had significantly greater carotid atherosclerosis. Among women who were carriers of the *APOE*ε4 genotype, those with higher PTSD symptoms had greater brain white matter hyperintensities, an indicator of brain small vessel disease, as well as poorer cognition.

**Meaning:**

These findings suggest that adverse implications of PTSD symptoms for both cardiovascular and neurocognitive health at midlife, particularly for women who are carriers of *APOE*ε4.

## Teaser

This cross-sectional study assesses associations of posttraumatic stress disorder (PTSD) symptoms with carotid intima media thickness and white matter hyperintensity volume among midlife women and by *APOE*ε4 status.

## Introduction

Cardiovascular disease (CVD) and Alzheimer disease (AD) are major women’s health issues. CVD is the leading cause of death among US women, with 45% of women developing CVD in their lifetime.^1^ AD is the fourth leading cause of death among US women.^2^ Furthermore, approximately two-thirds of individuals with AD and related disorders are women.^3^

An issue with relevance for cardiovascular and neurocognitive health is posttraumatic stress disorder (PTSD). Most women in the US will experience at least 1 major traumatic event in their life,^4^ and 10% will develop PTSD. Women have double the risk of PTSD relative to men.^5^ PTSD is associated with a 50% to 60% increased risk of incident CVD^6^ and elevated stroke^7^ and dementia^8^ risk.

While evolving literature links PTSD to women’s cardiovascular and neurocognitive health, key questions remain. First, the existing literature relies on male samples, with few studies in women and even fewer among midlife women. An exception is the Nurses’ Health Study II (NHS II) of midlife women, which found associations between increased PTSD symptoms and worse cognitive function assessed via a self-administered online cognitive battery.^9,10,11^ However, NHS II lacked vascular and brain health measures. Midlife is a critical time for women’s cardiovascular and brain health, as it occurs directly before the onset of clinical CVD^12^ and is when initial hallmarks of AD and related disorders (eg, amyloid β, hyperphosphorylated tau) begin.^13^ Midlife includes menopause, a time of accelerating vascular risk,^14^ decreased memory,^15^ and potential emergence of effects of earlier stress exposure.^16^ Second, although the interconnections between the heart and the brain are increasingly appreciated,^17^ few studies bridge these systems. Furthermore, few studies have considered a role for the *APOE*ε4 (OMIM 107741) genotype, a risk factor for poor cardiovascular health, cognitive decline, and dementia, particularly for women, and potentially pointing to women particularly vulnerable to neurocognitive and cardiovascular insults.^18,19^

We tested whether higher PTSD symptoms would be associated with higher carotid IMT, greater brain white matter hyperintensity (WMH) or volume (WMHV), and poorer cognition among midlife women who underwent vascular imaging, neuroimaging, and a comprehensive neuropsychological battery. Carotid IMT, or ultrasonographically assessed thickness of the intimal and medial layers of the carotid artery, is an established subclinical CVD indicator associated with future CVD events and useful for assessing cardiovascular health among midlife women among whom other subclinical indicators (eg, coronary calcification) may lack sensitivity.^20,21^ WMHs are lesions in the white matter apparent on magnetic resonance imaging (MRI) that reflect, in part, small vessel disease and are linked to later dementia, cognitive decline, and mortality.^22^ Collectively, IMT and WMHs help identify women at risk for future disease. Furthermore, we tested a modifying role of the *APOE*ε4 genotype, hypothesizing that women who were *APOE*ε4 carriers would be at particularly elevated cardiovascular and neurocognitive risk with PTSD symptoms.

## Methods

This cross-sectional study was reviewed and approved by the University of Pittsburgh Human Research Protection Office. Participants provided written informed consent. This study follows the Strengthening the Reporting of Observational Studies in Epidemiology (STROBE) reporting guideline for cross-sectional studies. Participants underwent screening procedures, physical measurements, medical history interview, questionnaires, phlebotomy, neuropsychological testing, carotid artery ultrasonography, and brain MRI.

### Sample

The MsBrain study included 274 women ages 45 to 67 years recruited in 2017 to 2020 for a study of menopause and brain health.^23^ Participants were recruited from the Pittsburgh, Pennsylvania, community via advertisements, registry mailings, and a menopause and cardiovascular health study.^24^ MsBrain exclusion criteria reflected the parent study on menopause and included pregnancy, hysterectomy or bilateral oophorectomy, history of stroke or cerebrovascular accident, Parkinson disease, current chemotherapy, history of dementia, seizure disorder, brain tumor, active substance abuse (established via urine toxicology screen), history of head trauma with loss of consciousness more than 60 minutes, contraindication to MRI, and use of systemic estrogen or progesterone, selective estrogen receptor modulators, aromatase inhibitors, gabapentin, selective serotonin reuptake inhibitors, or serotonin norepinephrine reuptake inhibitors.

Of 664 women screened, 274 women were eligible, enrolled, and underwent study procedures. The number of women in models varied by the outcome under study (IMT, WMHV, cognition). For IMT, 272 women underwent a carotid ultrasonographic examination, and since 2 women were missing phlebotomy, 270 women were included in models with blood biomarkers. For WMHV, 239 women underwent neuroimaging, 9 were excluded due to brain tumor, stroke, or seizure disorder detected, and 5 women were excluded due to a chemotherapy history, yielding 225 women included in analyses (223 women with blood biomarkers). For cognition, 9 women were excluded due to brain tumor, stroke, or seizure disorder, 1 woman was excluded due to tardive dyskinesia, and 8 women were excluded from select tests due to English language limitations, yielding 261 included in analyses. Finally, due to refusal of genetic testing, analyses incorporating *APOE* included 257 women for IMT, 215 women for WMHV, and 247 women for cognition. Women excluded from any model had a higher body mass index (BMI; calculated as weight in kilograms divided by height in meters squared) (31.25 vs 28.21; *t*_272_ = 2.9; *P* = .004) and were more likely to be Black (35.94% vs 11.90%; Cramer *V* = 0.26; *P* < .001) than women included in all models.

### Measures

#### PTSD Symptoms

The PTSD Checklist–Civilian Version (PCL-C)^25^ is a validated, 17-item self-report inventory assessing PTSD symptoms over the past month, with items rated from 1, indicating not at all, to 5, extremely, and higher scores indicating higher PTSD symptoms. Primary models considered the continuous summed score and secondary models categorized PTSD by the clinical cutoff (≥30).^26,27^

#### Carotid Ultrasonography

Certified sonographers at the University of Pittsburgh’s Ultrasound Research Laboratory obtained bilateral carotid images via B-mode ultrasonography using a Sonoline Antares (Siemens) high-resolution duplex scanner (VF10-5 transducer) according to a standardized protocol.^28^ Digitized images were obtained at end-diastole from 8 locations (4 locations from left and right carotid arteries): near and far walls of the distal common carotid artery, far walls of the carotid bulb, and the internal carotid artery. Images were read using semiautomated reading software. Values were obtained by electronically tracing the lumen-intima interface and the media-adventitia interface across a 1-cm segment for each segment. IMT was the mean of the mean readings across the 8 locations. Reproducibility was excellent (intraclass correlation coefficient: between sonographers, ≥0.87; between readers, 0.92).

#### WMHV

MRI scanning was performed at the MR Research Center of the University of Pittsburgh with a 3T Siemens Tim Trio MR scanner and a Siemens 64-channel head coil.^23^ MRIs were magnetization-prepared rapid gradient echo (MPRAGE) T1-weighted sequence and T2-weighted (T_2_w) fluid-attenuated inversion recovery (FLAIR) sequence. MPRAGE images were acquired in the axial plane (parameters: repetition time, 2400 ms; echo time, 2.22 ms; T1, 1000 ms; flip angle, 8°; field of view, 256 × 240 mm; slice thickness, 0.8 mm; voxel size, 0.8 mm × 0.8mm; matrix size, 320 × 300; number of slices, 208). FLAIR images were acquired in the axial plane (parameters: repetition time, 9690 or 10 000 ms; echo time, 91 ms; T1, 2500 ms; flip angle, 135°; field of view, 256 × 256 mm; matrix, 320 × 320; slice thickness, 1.6 mm; voxel size, 0.8 mm × 0.8 mm; number of slices, 104).

An automated pipeline was used to segment WMH on the T_2_w FLAIR images using previously validated methods.^29^ Cerebral and cerebellar white matter were segmented on the T_1_w image and mapped into the T_2_w FLAIR image space using SPM mapping software version 12 (Functional Imaging Laboratory, UCL Queen Square Institute of Neurology) and FreeSurfer processing, analyzing, and visualizing software version 7.1.1 (Athinoula A. Martinos Center for Biomedical Imaging, Harvard Medical School). Cerebellar white matter represented normal-appearing white matter; its intensity mean and SD were used for *Z*-transformation of the T_2_w FLAIR image. A threshold of 2 was applied on *Z*-transformed FLAIR images. *Z*-transformation also reduces intensity variations across individual FLAIR images.

In the processing, analyzing, and visualizing software, white matter was parcellated according to its nearest cortex with the Deskian-Killiany atlas, used to generate the cortical white matter masks for frontal, temporal, parietal, and occipital lobes for localization of WMHs. White matter parcellations corresponding to frontal cortex regions were combined to create a frontal cortical white matter mask to localize frontal WMHs. Cortical white matter masks were generated for temporal, parietal, and occipital lobes. These lobular cortical white matter masks did not overlap and were combined to create an overall cortical and deep white matter mask. White matter surrounding the ventricles that was not part of the cortical and deep white matter mask comprised the periventricular white matter mask. The total and regional WMHV (in centimeters cubed) were normalized as WMH divided by intracranial volume and log transformed.

#### Cognitive Performance

Trained and certified testers administered an in-person neuropsychological battery. Participants were tested for attention and working memory using the Letter-Number Sequencing,^30^ control and experimental versions. Processing speed was tested using the Symbol Digit Modalities Test.^31^ Participant perceptual speed was tested using Finding A’s.^32^ Memory was assessed using the California Verbal Learning Test-2^33^ short and long-delay free recall, and learning was tested using the California Verbal Learning Test-2 total score on trials 1 to 5. We assessed letter fluency using the Letter Fluency Test PRW set.^34^ Semantic fluency was assessed with the animals task.^35^ Spatial ability was assessed with the Card Rotations Test.^32^ Finally, global cognitive function was assessed with the Montreal Cognitive Assessment.^36^

#### Additional Measures

Height and weight were measured via standard methods and BMI was calculated. Systolic blood pressure (SBP) and diastolic blood pressure (DBP) were the mean of 3 seated measurements. Demographics, medical history, and medication use were assessed by questionnaires and interview. Race, ethnicity, gender, and education (years of education) were self-reported. Race and ethnicity were assessed because due to previously documented differences in PTSD, cardiovascular, and neurocognitive risk. Race and ethnicity were categorized as Asian, Black, White, or multiracial. Head injury history was assessed. Current and lifetime substance use (eg, amphetamines, opiates, hallucinogens, benzodiazepines, marijuana) was assessed via urine toxicology screen and questionnaire. Physical activity was assessed using the International Physical Activity Questionnaire,^37^ and depressive symptoms were assessed with the Center for Epidemiologic Studies of Depression scale.^38^

Women underwent phlebotomy after overnight fast. Glucose, total cholesterol, high density lipoprotein (HDL) cholesterol, and triglycerides were determined using enzymatic assays and insulin via immunoturbidimetric assay. Low-density lipoprotein (LDL) cholesterol was calculated via the Friedewald equation.^39^ Homeostatic model assessment (HOMA) for insulin resistance was calculated as (insulin × glucose) / 22.5. Genotypes for *APOE* polymorphisms, rs429358 (*APOE*ε4), and rs7412 (*APOE*ε2) were determined using TaqMan genotyping assays (Thermo Fisher Scientific).^40^ Because of the strong linkage disequilibrium between sites, this is also treated as a 3-allele *APOE* polymorphism: *APOE*ε2, *APOE*ε3, and *APOE*ε4, yielding 6 genotypes (ε2/ε2, ε2/ε3, ε2/ε4, ε3/ε3, ε3/ε4, ε4/ε4). Participants were classified as *APOE*ε4 carriers (ε2/ε4, ε3/ε4, ε4/ε4) or not (ε2/ε2, ε2/ε3, ε3/ε3).

### Statistical Analysis

PTSD symptoms, IMT, BMI, HOMA, triglycerides, physical activity, and WMHV were log transformed. We tested our primary aims in separate linear regression models testing associations of PTSD symptoms with IMT, PTSD symptoms with whole-brain WMHV, and PTSD symptoms with cognitive performance. Regional WMHV were considered secondarily. Covariates in models of IMT or WMHV were age, race and ethnicity, education, BMI, SBP, HOMA, HDL, triglycerides, smoking, physical activity, and use of BP-lowering, diabetes, and lipid medications. While covariates were selected in an a priori manner, 1 BP variable (SBP) and HDL and triglycerides were included in models, given collinearity between BP variables and between lipid variables, respectively. Covariates for models of cognitive performance were age, race and ethnicity, and education. Interactions between PTSD and *APOE*ε4 in relation to study outcomes (IMT, WMH, cognition) were tested in separate models; where there was a significant interaction, models were stratified by *APOE*ε4 status. Given the number of cognitive tests administered, in additional cognitive outcome models, we used the false discovery rate method to account for multiple comparisons.^41^ In sensitivity analyses, we considered associations of PTSD with IMT, WMH, or cognition also covarying for substance use, head injury history, or depressive symptoms (given the high correlation between depressive and PTSD symptoms [*r* = 0.67], a residualized depressive symptom score was considered). In additional secondary models, we tested an indirect effect of IMT in the association of PTSD symptoms with WMHV or cognition via Sobel tests. We similarly considered an indirect effect of WMHV in associations of PTSD symptoms with cognition in separate models. For all models, diagnostic statistics and graphical outputs were examined to assess model assumptions, model fit, and influence of individual data points. Tests were 2-tailed with α = .05. Analyses were conducted in SAS software version 9.4 (SAS Institute). Data were analyzed from July 2022 to September 2023.

## Results

Among 274 participants (mean [SD] age, 59.03 [4.34] years; 6 Asian participants [2.2%]; 48 Black participants [17.5%]; 215 White participants [78.5%]; 5 multiracial participants [1.8%]), 64 participants (24.71%) were *APOE*ε4 genotype carriers. Participants had a median (IQR) BMI of 27.36 (23.99-32.68), and mean (SD) SBP was 118.93 (15.06) mm Hg and DBP was 68.52 (8.90) mm Hg (Table 1).

**Table 1.** Participant Characteristics

Characteristic	Participants, No. (%) (N = 274)
Age, mean (SD), y	59.03 (4.34)
Race and ethnicity	
Asian	6 (2.19)
Black	48 (17.52)
White	215 (78.47)
Multiracial	5 (1.82)
Years of education, mean (SD), y	15.67 (2.43)
BMI, median (IQR)	27.36 (23.99-32.68)
BP, mean (SD), mm Hg	
Systolic	118.93 (15.06)
Diastolic	68.52 (8.90)
Cholesterol, mean (SD), mg/dL	
LDL	120.52 (34.84)
HDL	69.93 (20.39)
Triglycerides, median (IQR), mg/dL	92.50 (70.00-122.50)
HOMA, median (IQR)	2.93 (1.39-4.35)
Current smoking	5 (1.82)
Leisure time physical activity, median (IQR)^a^	594 (99.00-1611.00)
Medication use	
BP-lowering	53 (19.34)
Diabetes	12 (4.38)
Lipid	41 (14.96)
*APOE*ε4 carrier	64 (24.71)
PTSD symptoms, median (IQR)^b^	22.00 (19.00-28.00)
Depressive symptoms, median (IQR)^c^	6.00 (3.00-11.00)
IMT, median (IQR), mm	0.70 (0.63-0.77)
WMHV, median (IQR)^d^	
Whole brain	0.065 (0.043-0.102)
Deep	0.013 (0.007-0.026)
Periventricular	0.050 (0.033-0.078)
Frontal	0.003 (0.002-0.006)
Parietal	0.001 (0.0001-0.003)
Temporal	0.003 (0.001-0.006)
Occipital	0.003 (0.001-0.009)

Abbreviations: BMI, body mass index (calculated as weight in kilograms divided by height in meters squared); BP, blood pressure; HDL, high-density lipoprotein; HOMA, homeostatic model assessment; IMT, intima media thickness; LDL, low-density lipoprotein; PTSD, posttraumatic stress disorder; WMHV, white matter hyperintensity volume.

SI conversion factors: To convert cholesterol to millimoles per liter, multiply by 0.0259; to convert triglycerides to millimoles per liter, multiply by 0.0113.

^a^ Measured using the International Physical Activity Questionnaire (higher score indicates more and/or more vigorous physical activity).

^b^ Measured using the PCL-C, Post Traumatic Stress Disorder Checklist, Civilian Version (range, 17-85; higher score indicates greater symptom severity).

^c^ Measured using the Center for Epidemiologic Studies of Depression Scale (range, 0-60; higher score indicates greater depressive symptoms).

^d^ WMHV expressed as centimeters cubed divided by intracranial volume.

We first examined the IMT. Women with greater PTSD symptoms had higher carotid IMT (Table 2), and associations persisted controlling for CVD risk factors. Associations between PTSD and IMT did not significantly vary by *APOE*ε4 status (*P* for interaction = .71).

**Table 2.** Associations Between PTSD Symptoms and Carotid IMT

Measure	IMT, β (95%CI)	
	Model 1 (n = 272)^a^	Model 2 (n = 270)^b^
PTSD symptoms	0.07 (0.01 to 0.13)^c^	0.07 (0.01 to 0.13)^c^

Abbreviations: IMT, intima media thickness; PTSD, posttraumatic stress disorder.

^a^ Adjusted for age, race, education, and log of body mass index.

^b^ Adjusted for variables in model 1, plus systolic blood pressure, log of homeostatic model assessment, high-density lipoprotein cholesterol, log triglycerides, smoking status, log of physical activity score, and use of blood pressure–lowering medications, diabetes medications, and lipid medications.

^c^ *P* < .05; PTSD symptoms and IMT log transformed.

**Figure 1.**
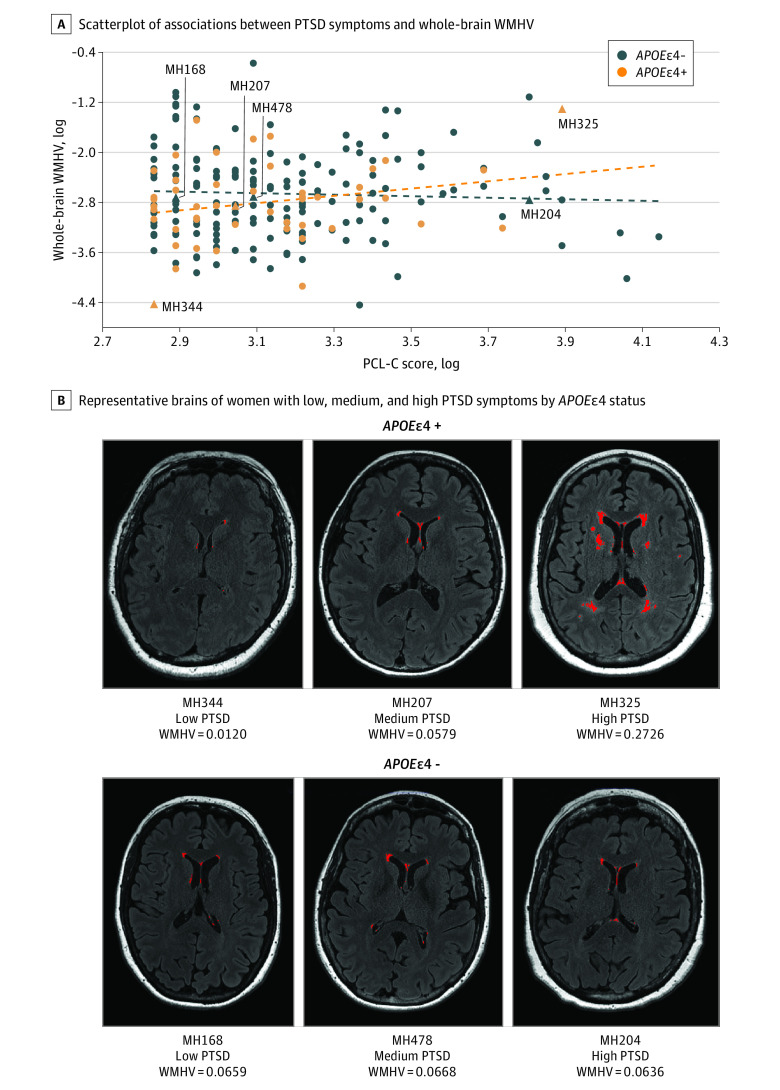
Associations Between Posttraumatic Stress Disorder (PTSD) Symptoms and White Matter Hyperintensity Volume (WMHV) For anonymity, patients are identified with MH numbers, eg, MH168. − indicates noncarrier; +, carrier; PCL-C, PTSD Checklist–Civilian Version.

In the WMHV models, interactions between PTSD symptoms and *APOE*ε4 status were observed in the primary outcome of whole-brain WMHV (*P* for interaction = .02), as well as periventricular (*P* for interaction = .03) and parietal WMHV (*P* for interaction = .03) in multivariable models. Among women who were *APOE*ε4 carriers, PTSD symptoms were associated with greater whole-brain (β = 0.96 [95% CI, 0.30 to 1.63]; *P* = .009), periventricular (β = 0.90 [95% CI, 0.24 to 1.56]; *P* = .02), deep (β = 1.21 [95% CI, 0.23 to 2.20]; *P* = .01), and frontal (β = 1.25 [95% CI, 0.05 to 2.45]; *P* = .04) WMHV (Table 3 and Figure 1).

**Table 3.** Association of Posttraumatic Stress Disorder Symptoms With WMHV by *APOE*ε4 Status

*APOE*ε4 status	WMHV, β (95%CI)^a^						
	Whole brain	Periventricular	Deep	Frontal	Parietal	Occipital	Temporal
Carrier (n = 48)	0.96 (0.30 to 1.63)^b^	0.90 (0.24 to 1.56)^c^	1.21 (0.23 to 2.20)^c^	1.25 (0.05 to 2.45)^c^	1.70 (−0.34 to 3.74)	1.26 (−0.74 to 3.25)	1.14 (−0.33 to 2.60)
Noncarrier (n = 167)	−0.07 (−0.44 to 0.29)	0.01 (−0.35 to 0.37)	−0.15 (−0.72 to 0.42)	0.04 (−0.63 to 0.70)	−0.66 (−1.67 to 0.35)	−0.63 (−1.50 to 0.25)	−0.15 (−0.85 to 0.55)

Abbreviation: WMHV, white matter hyperintensity volume.

^a^ PTSD symptoms and WMHV values log transformed. Analyses were adjusted for age, race, education, log body mass index, smoking, systolic blood pressure, log of homeostatic model assessment, high-density lipoprotein cholesterol, log triglycerides, smoking status, log of physical activity score, and use of blood pressure–lowering medications, diabetes medications, and lipid medications.

^b^ *P* < .01.

^c^ *P* < .05.

**Figure 2.**
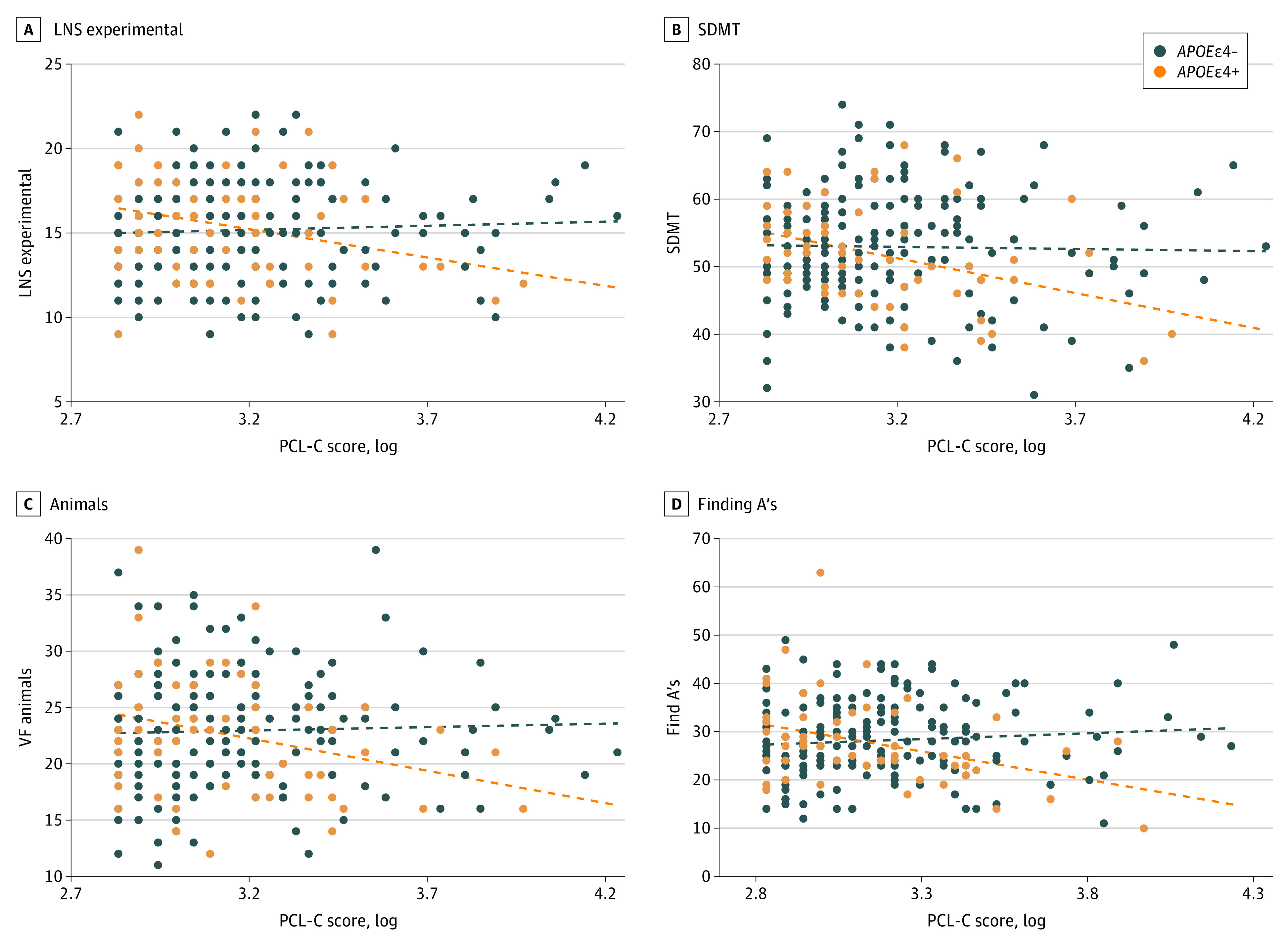
Scatterplots of Associations Between Posttraumatic Stress Disorder (PTSD) Symptoms and Select Neuropsychological Tests − indicates noncarrier; +, carrier; LNS, Letter-Number Sequencing (experimental); PCL-C, PTSD Checklist Civilian Version; SDMT, Symbol Digit Modalities Test; VF, verbal fluency.

We observed significant interactions between PTSD symptoms and *APOE*ε4 status in association with cognitive outcomes, specifically attention and working memory, semantic fluency, processing speed, and perceptual speed. Among women who were *APOE*ε4 carriers, PTSD symptoms were associated with poorer attention and working memory (β = −3.37 [95% CI, −6.12 to −0.62]; *P* = .02), semantic fluency (β = −6.01 [95% CI, −10.70 to −1.31]; *P* = .01), processing speed (β = −11.05 [95% CI, −17.80 to −4.30]; *P* = .002), and perceptual speed (β = −12.73 [95% CI, −20.71 to −4.75]; *P* = .002) (Figure 2; eTable 1 in Supplement 1).

In secondary models, given the number of neuropsychological tests considered, associations between PTSD and cognition were tested controlling for multiple comparisons. Findings for processing speed and perceptual speed remained among women who were *APOE*ε4 carriers (eTable 1 in Supplement 1). We also considered associations of PTSD with outcomes using the PCL-C clinical cutoff; 51 women (19%) scored in this range. Whereas clinically elevated PTSD symptoms were not significantly associated with IMT, among women who were *APOE*ε4 carriers, PTSD symptoms were associated with higher whole-brain WMHV, deep WMHV, and poorer processing speed and perceptual speed (eTables 2-4 in Supplement 1). Furthermore, additionally covarying for depressive symptoms, history of head injury, or substance use history did not account for associations of PTSD with IMT, WMHV, or cognition (eTables 5-7 in Supplement 1). Finally, there was no evidence of an indirect effect of IMT in associations between PTSD and WMHV (whole-brain WMHV: β = 0.12 [95% CI, −0.10 to 0.35]; *P* = .29), nor any indirect effects of IMT (eg, processing speed: β = 0.20 [95% CI, −0.76 to 1.16]; *P* = .68) or whole-brain WMHV (eg, processing speed: β = 0.07 [95% CI, −2.92 to 3.06]; *P* = .96) in associations of PTSD with cognitive performance among women who were *APOE*ε4 carriers.

## Discussion

In this cross-sectional study among midlife women, higher PTSD symptoms were associated with greater carotid atherosclerosis. Furthermore, among women who were *APOE*ε4 carriers, PTSD symptoms were associated with greater WMHV (whole brain, periventricular, deep, frontal) and poorer cognitive performance across multiple domains. These findings point to the adverse outcomes associated with PTSD symptoms for cardiovascular and neurocognitive health at midlife, particularly for women who are *APOE*ε4 carriers.

Some prior studies, largely focused on men and/or veterans, have considered PTSD symptoms in relation to cardiovascular or brain health. For IMT, some studies have reported that PTSD is associated with higher IMT, yet findings are mixed and focused on male veterans or specific populations (eg, adults experiencing deportation).^42,43^ Some studies have indicated associations of PTSD symptoms with WMH^44^ and cognition,^45^ yet these studies relied on data from males, veterans, and/or individuals undergoing PTSD treatment. The NHS II of midlife women found that women with histories of trauma exposure and more PTSD symptoms had greater declines in cognitive performance over approximately 1 year compared with women without PTSD symptoms.^10^ However, in the NHS II study, cognitive performance was assessed via a brief online battery assessing 2 domains: learning and working memory and psychomotor speed and attention. Our study is notable for its assessment of cognition via a comprehensive, in-person neuropsychological test battery assessing a range of domains. Collectively, this study underscores the sensitivity of working memory, psychomotor speed, and perceptual speed and visual attention to PTSD symptoms. MsBrain also shows the importance of *APOE*ε4 genotype status in these associations. Thus, this study sheds important insight on the implications of PTSD symptoms to women’s cardiovascular and neurocognitive health.

Our study is notable in considering the *APOE*ε4 genotype, which is associated with dementia risk, particularly in women.^18^ Prior studies have also found that the *APOE*ε4 genotype is associated with elevated CVD and PTSD risk,^19,46,47^ and studies with male veterans considered *APOE*ε4 in associations of PTSD to cognition.^48,49^ In our study, *APOE*ε4 genotype was an important modifier of associations of PTSD with neurocognitive outcomes but not carotid atherosclerosis, perhaps due to the greater potency of the *APOE*ε4 genotype in neurocognitive risk.^18^ Our findings indicate that the *APOE*ε4 genotype may identify a group of women with PTSD symptoms at particular risk for poor neurocognitive health.

We considered PTSD symptoms in association with the regional distribution of WMHV. Women who were *APOE*ε4 carriers who had higher PTSD symptoms had greater whole-brain WMHV, periventricular WMHV, deep WMHV, and WMHV in the frontal lobe. Notably, frontal lobe WMHs have been particularly linked to vascular risk,^50^ suggesting the importance of vascular processes here.

The potential mechanisms linking PTSD symptoms to cardiovascular and neurocognitive health are multiple. PTSD has been associated with poorer CVD risk factors, which contribute to the development of vascular disease,^51^ yet we controlled for these factors. We considered education, depressive symptoms, head injury, and substance use, which did not explain associations. PTSD symptoms have been associated with altered emotion processing and neural circuitry implicated in cognition^45^ and stressor-induced cardiovascular reactivity.^52^ Other potential pathways, such as inflammatory, autonomic, hypothalamic pituitary adrenal, or epigenetic processes, warrant future consideration.

This study has several strengths. It included a large, well-characterized community sample of midlife women. It leveraged vascular and neuroimaging, providing subclinical indicators of peripheral vascular and cerebrovascular health decades before clinical disease is present. A comprehensive neuropsychological battery was performed. *APOE* genotyping was conducted, showing pronounced modification by *APOE*ε4 status. The interconnections between the cardiovascular system and brain are increasingly appreciated; this study is unique in considering them together, showing implications of PTSD across systems.

### Limitations

This study has some limitations. We did not conduct diagnostic clinical interviews, nor did we assess PTSD treatment. Study exclusions included common antidepressants, head injury, active substance use, and several cardiovascular and neurological diseases. Thus, participants were likely less distressed and healthier than the general population, which may have restricted the range on certain indicators. Participants had relatively low levels of PTSD symptoms, yet it is notable that associations were observed even at these low levels. To measure PTSD symptoms, we used the PCL-C, based on *Diagnostic and Statistical Manual of Mental Disorders* (Fourth Edition) PTSD criteria, rather than measures (eg, PCL-5) based on *Diagnostic and Statistical Manual of Mental Disorders* (Fifth Edition) criteria. However, there is high agreement between PCL-C and PCL-5.^53^ Women excluded from any model had a higher BMI and were more often Black than women included in all models. Furthermore, all participants identified as cisgender, and most were non-Hispanic Black or White. Results may not generalize to all groups. Future work should consider more diverse samples. Given the multiple tests conducted, results, particularly for secondary findings, should be regarded with caution. The study was observational and cross-sectional; we cannot make assertions about directionality or causality.

## Conclusions

The findings of this cross-sectional study underscore the important implications of PTSD and its symptoms for women’s cardiovascular and brain health, with women who were *APOE*ε4 carriers particularly at risk. PTSD is a major women’s health issue, affecting 10% of women in their lifetime. Our findings point to an at-risk population that may warrant early intervention and prevention efforts to reduce cardiovascular and neurocognitive risk at midlife and beyond.

## Notes

Supplement 1. **eTable 1.** Association of PTSD Symptoms to Cognitive Performance, by *APOE*ε4 Status **eTable 2.** Association of Clinically-Elevated PTSD Symptoms to IMT **eTable 3.** Clinically-Elevated PTSD Symptoms in Relation to WMHV, by *APOE*ε4 Status **eTable 4.** Association of Clinical PTSD Symptoms to Cognitive Performance, by *APOE*ε4 Status **eTable 5.** Association of PTSD Symptoms to IMT, Additional Covariates **eTable 6.** PTSD Symptoms in Relation to WMHV Among *APOE*ε4 Positive Women, Additional Covariates **eTable 7.** PTSD Symptoms in Relation to Cognition Among *APOE*ε4 Positive Women, Additional Covariates

Supplement 2.
Data Sharing Statement
