# Supplementary material for: Posttraumatic Stress Disorder Symptoms and Cardiovascular and Brain Health in Women
Source: JAMA Netw Open. 2023 Nov 2;6(11):e2341388. doi: 10.1001/jamanetworkopen.2023.41388 (PMC10623197; doi:10.1001/jamanetworkopen.2023.41388)
Supplement: Supplement 1. — eTable 1. Association of PTSD Symptoms to Cognitive Performance, by APOEε4 Status eTable 2. Association of Clinically-Elevated PTSD Symptoms to IMT eTable 3. Clinically-Elevated PTSD Symptoms in Relation to WMHV, by APOEε4 Status eTable 4. Association of Clinical PTSD Symptoms to Cognitive Performance, by APOEε4 Status eTable 5. Association of PTSD Symptoms to IMT, Additional Covariates eTable 6. PTSD Symptoms in Relation to WMHV Among APOEε4 Positive Women, Additional Covariates eTable 7. PTSD Symptoms in Relation to Cognition Among APOEε4 Positive Women, Additional Covariates [file jamanetwopen-e2341388-s001.pdf]

Supplemental Online Content

Thurston RC, Jakubowski K, Chang Y, et al. Posttraumatic stress disorder symptoms and cardiovascular and brain health in women. *JAMA Netw Open*. 2023;6(11):e2341388. doi:10.1001/jamanetworkopen.2023.41388

eTable 1. Association of PTSD Symptoms to Cognitive Performance, by *APOE*ε4 Status
eTable 2. Association of Clinically-Elevated PTSD Symptoms to IMT
eTable 3. Clinically-Elevated PTSD Symptoms in Relation to WMHV, by *APOE*ε4 Status
eTable 4. Association of Clinical PTSD Symptoms to Cognitive Performance, by *APOE*ε4 Status
eTable 5. Association of PTSD Symptoms to IMT, Additional Covariates
eTable 6. PTSD Symptoms in Relation to WMHV Among *APOE*ε4 Positive Women, Additional Covariates
eTable 7. PTSD Symptoms in Relation to Cognition Among *APOE*ε4 Positive Women, Additional Covariates

This supplemental material has been provided by the authors to give readers additional information about their work.

© 2023 Thurston RC et al. *JAMA Network Open.*

eTable 1. Association of PTSD symptoms to cognitive performance, by *APOE4* status

	PTSD Symptoms			
	APOEε4 Positive		*APOEε4 Negative*	
Outcome	B	(95%CI)	B	(95%CI)
Learning and Memory (CVLT-2)				
Learning (across five trials)	-7.27	(-16.68, 2.14)	-0.68	(-4.86, 3.49)
Short delay free recall	-1.80	(-5.00, 1.39)	0.62	(-.74, 1.97)
Long delay free recall	-1.82	(-4.74, 1.11)	0.43	(-.88, 1.74)
Attention/working memory (LNS)				
Experimental	-3.37	(-6.12, -.62)*#	0.46	(-.87, 1.78)
Control	-1.07	(-4.02, 1.87)	0.97	(-.71, 2.65)
Processing speed (SDMT)	-11.05	(-17.80, -4.30)**¶	-1.17	(-5.00, 2.66)
Perceptual speed (Finding A's)	-12.73	(-20.71, -4.75)**¶	2.32	(-1.63, 6.27)
Letter fluency (PRW)	-9.06	(-19.81, 1.69)†	-0.78	(-5.98, 4.41)
Semantic fluency (Animals)	-6.01	(-10.70, -1.31)*#	0.64	(-1.71, 2.99)
Global cognition (MoCA)	-0.20	(-2.40, 1.99)	0.30	(-.83, 1.42)

†p<.10, *p<.05, **p<.01; with False Discovery Rate correction: #p<.10, ¶p<.05

Note: PTSD symptoms log transformed

Covariates: age, race, years of education

*APOE*ε4 positive: N=61, *APOE*ε4 negative: N=186

© 2023 Thurston RC et al. *JAMA Network Open.*

eTable 2. Association of clinically-elevated PTSD symptoms to IMT

	IMT			
	Model 1		Model 2	
	B	(95%CI)	B	(95%CI)
Clinical PTSD symptoms	.03	(-.01, .08)	.03	(-.01, .07)

Note: IMT log transformed; clinical PTSD symptoms: PCL-C scores ≥30

Model 1: N=272; Models 2: N=270

Model 1: Age, race, education, BMI (log)

Model 2: + SBP, HOMA (log), HDL, triglycerides (log), smoking, physical activity (log), BP-lowering medications, diabetes medications, lipid medications

© 2023 Thurston RC et al. *JAMA Network Open.*

eTable 3. Clinically-elevated PTSD symptoms in relation to WMHV, by *APOE*ε4 status

	WMHV						
	Whole Brain	Peri-ventricular	Deep	Frontal	Parietal	Occipital	Temporal
	B (95%CI)	B (95%CI)	B (95%CI)	B (95%CI)	B (95%CI)	B (95%CI)	B (95%CI)
*APOE*ε4 *positive*							
Clinical PTSD symptoms	0.59 (0.06, 1.12)*	0.54 (-0.26, 1.34)	0.60 (0.08, 1.11)*	0.59 (-0.36, 1.55)	0.75 (-0.79, 2.28)	0.89 (-0.70, 2.48)	0.42 (-0.73, 1.56)
*APOE*ε4 *negative*							
Clinical PTSD symptoms	0.12 (-0.13, 0.38)	0.10 (-.031, 0.51)	0.16 (-0.09, 0.42)	0.26 (-0.22, 0.73)	-0.01 (-0.64, .62)	0.07 (-0.65, .80)	0.03 (-0.48, 0.53)

†p<.10, *p<.05, **p<.01; *Note*: WMHV values log transformed; Clinical PTSD symptoms: PCL-C scores ≥30

Adjusted for age, race, education, BMI (log), smoking, SBP, HOMA (log), triglycerides (log), HDL, physical activity, BP-lowering medications, lipid medications, diabetes medications

*APOE*ε4 positive: N=48, *APOE*ε4 negative: N=167

© 2023 Thurston RC et al. *JAMA Network Open.*

eTable 4. Association of clinical PTSD symptoms to cognitive performance, by *APOE4* status

	Clinical PTSD Symptoms			
	APOEε4 Positive		*APOEε4 Negative*	
Outcome	B	(95%CI)	B	(95%CI)
Learning and Memory (CVLT-2)				
Learning (across five trials)	-3.92	(-10.80, 2.96)	-0.35	(-3.44, 2.75)
Short delay free recall	-0.29	(-2.62, 2.05)	0.31	(-0.69, 1.31)
Long delay free recall	-0.61	(-2.76, 1.53)	0.28	-0.69, 1.24)
Attention/working memory (LNS)				
Experimental	-1.44	(-3.51, 0.62)	0.10	(-0.87, 1.08)
Control	-0.41	(-2.55, 1.72)	0.12	(-1.12, 1.36)
Processing speed (SDMT)	-6.89	(-11.92, -1.86)**	-2.82	(-5.62, -0.03)*
Perceptual speed (Finding A's)	-7.21	(-13.28, -1.15)*	-0.52	(-3.43, 2.39)
Letter fluency (PRW)	-3.66	(-11.58, 4.26)	-3.88	(-7.68, -0.08)*
Semantic fluency (Animals)	-3.37	(-6.85, 0.11)	0.72	(-1.02, 2.46)
Global cognition (MoCA)	-0.13	(-1.70, 1.43)	-0.37	(-1.20, 0.46)

†p<.10, *p<.05, **p<.01

Note: Clinical PTSD symptoms: PCL-C scores ≥30

Covariates: age, race, years of education

APOEε4 positive: N=61, *APOEε4* negative: N=186

© 2023 Thurston RC et al. *JAMA Network Open.*

eTable 5. Association of PTSD symptoms to IMT, additional covariates

	IMT					
	Model 1		Model 2		Model 3	
	B	(95%CI)	B	(95%CI)	B	(95%CI)
PTSD symptoms	0.07	(0.01, 0.13)*	0.07	(0.01, 0.13)*	0.06	(0.003, 0.12)*

†p<.10, *p<.05, **p<.01

Note: IMT and PTSD symptoms log transformed

Model 1: Age, race, education, BMI (log), SBP, HOMA (log), HDL, triglycerides (log), smoking, physical activity (log), BP-lowering medications, diabetes medications, lipid medications, depressive symptoms

Model 2: Age, race, education, BMI (log), SBP, HOMA (log), HDL, triglycerides (log), smoking, physical activity (log), BP-lowering medications, diabetes medications, lipid medications, history of head injury

Model 3: Age, race, education, BMI (log), SBP, HOMA (log), HDL, triglycerides (log), smoking, physical activity (log), BP-lowering medications, diabetes medications, lipid medications, substance use history

© 2023 Thurston RC et al. *JAMA Network Open.*

eTable 6. PTSD symptoms in relation to WMHV among *APOE*ε4 positive women, additional covariates

	WMHV						
	Whole Brain<br>B<br>(95%CI)	Periventricular<br>B<br>(95%CI)	Deep<br>B<br>(95%CI)	Frontal<br>B<br>(95%CI)	Parietal<br>B<br>(95%CI)	Occipital<br>B<br>(95%CI)	Temporal<br>B<br>(95%CI)
PTSD Symptoms							
Model 1	0.92	0.90	1.00	1.20	1.45	1.00	0.93
	(0.22, 1.61)*	(0.21, 1.60)*	(0.01, 1.98)*	(-0.06, 2.45)†	(-0.65, 3.56)	(-1.05, 3.06)	(-0.57, 2.43)
Model 2	1.04	0.97	1.29	1.39	2.03	1.23	1.36
	(0.35, 1.73)**	(0.29, 1.66)**	(0.26, 2.32)*	(0.14, 2.63)*	(-0.05, 4.11)	(-0.86, 3.33)	(-0.14, 2.86)
Model 3	0.96	0.91	1.18	1.24	1.59	1.26	1.06
	(0.28, 1.64)**	(0.28, 1.58)*	(0.28, 2.18)*	(0.28, 2.47)*	(1.28, 3.62)	(3.28, 3.30)	(2.28, 2.52)

†p<.10, *p<.05, **p<.01

Note: PTSD symptoms log transformed

Model 1: Age, race, education, BMI (log), SBP, HOMA (log), HDL, triglycerides (log), smoking, physical activity (log), BP-lowering medications, diabetes medications, lipid medications, depressive symptoms

Model 2: Age, race, education, BMI (log), SBP, HOMA (log), HDL, triglycerides (log), smoking, physical activity (log), BP-lowering medications, diabetes medications, lipid medications, history of head injury

© 2023 Thurston RC et al. *JAMA Network Open.*

Model 3: Age, race, education, BMI (log), SBP, HOMA (log), HDL, triglycerides (log), smoking, physical activity (log), BP-lowering medications, diabetes medications, lipid medications, substance use history

eTable 7. PTSD symptoms in relation to cognition among *APOEε*4 positive women, by *APOEε*4 status, additional covariates

	PTSD Symptoms					
	Model 1		*Model 2*		*Model 3*	
Outcome	B	(95%CI)	B	(95%CI)	B	(95%CI)
Learning and Memory (CVLT-2)						
Learning (across five trials)	-7.50	(-17.33, 2.33)	-7.24	(-16.75, 2.28)	-8.07	(-17.62, 1.49)†
Short delay free recall	-1.94	(-5.27, 1.39)	-1.80	(-5.03, 1.43)	-1.97	(-5.23, 1.30)
Long delay free recall	-2.27	(-5.28, 0.75)	-1.79	(-4.74, 1.16)	-2.15	(-5.10, 0.80)
Attention/working memory (LNS)						
Experimental	-3.68	(-6.53, -0.82)*	-3.34	(-6.11, -0.57)*	-3.33	(-6.15, -0.51)*
Control	-1.75	(-4.74, 1.23)	-1.03	(-4.00, 1.93)	-1.17	(-4.19, 1.84)
Processing speed (SDMT)	-10.71	(-17.76, -3.66)*	-11.00	(-17.81, -4.18)*	-11.37	(-18.31, -4.42)*
Perceptual speed (Finding A's)	-13.25	(-21.56, -4.95)*	-12.82	(-20.83, -4.81)*	-12.43	(-20.61, -4.26)*
Letter fluency (PRW)	-10.84	(-21.90, 0.22)†	-8.81	(-19.55, 1.94)	-9.70	(-20.67, 1.26)†
Semantic fluency (Animals)	-7.62	(-12.20, -3.04)*	-6.14	(-10.81, -1.46)*	-5.96	(-10.77, -1.14)*
Global cognition (MoCA)	-0.40	(-2.65, 1.85)	-0.21	(-2.43, 2.00)	-0.30	(-2.54, 1.94)

© 2023 Thurston RC et al. *JAMA Network Open.*

†p<.10, *p<.05, **p<.01

Note: PTSD symptoms log transformed

Model 1: Age, race, education, depressive symptoms

Model 2: Age, race, education, history of head injury

Model 3: Age, race, education, substance use history

© 2023 Thurston RC et al. *JAMA Network Open.*
