# Supplementary material for: Posttraumatic Stress Disorder Symptoms and Cardiovascular and Brain Health in Women
Source: JAMA Netw Open. 2023 Nov 2;6(11):e2341388. doi: 10.1001/jamanetworkopen.2023.41388 (PMC10623197; doi:10.1001/jamanetworkopen.2023.41388)
Supplement: Supplement 2. — Data Sharing Statement [file jamanetwopen-e2341388-s002.pdf]

Data Sharing Statement

Thurston. Posttraumatic Stress Disorder Symptoms and Cardiovascular and Brain Health in Women. *JAMA Netw Open*. Published November 03, 2023.
doi:10.1001/jamanetworkopen.2023.41388

Data
Data available: Yes
Data types: Deidentified participant data
How to access data: Requests should be made to thurstonrc@upmc.edu
When available: With publication

Supporting Documents
Document types: None

Additional Information
Who can access the data: The data and associated materials that support the findings of this study are available from the corresponding author upon reasonable request.
Types of analyses: For non-commercial use
Mechanisms of data availability: Approval of proposal with a signed data access agreement.
Any additional restrictions: Non-commercial use only.
